# Editorial: Expert opinion in bone research: 2021

**DOI:** 10.3389/fendo.2022.1071125

**Published:** 2022-11-16

**Authors:** Subhashis Pal

**Affiliations:** ^1^ Division of Endocrinology, Metabolism and Lipids, Department of Medicine, Emory University, Atlanta, GA, United States; ^2^ Emory Microbiome Research Center, Emory University, Atlanta, GA, United States

**Keywords:** postmenopausal osteoporosis, glucocorticoid induced osteoporosis, diabetic osteoporosis, vitamin D supplementation, Dieckol, linagliptin, kaempferol, tea extract

## Introduction

The current Research Topic on “*Expert opinion in bone research: 2021*” covers research in the area of emerging osteoporosis therapy, consisting of 4 current research contributed by 22 authors around the world. As an editor, I believe these articles will contribute largely to developing a successful osteoporosis treatment strategy. In this editorial, I summarize the main finding of these articles and their potential impact on the field of science.

## Use of tea extract for postmenopausal osteoporosis therapy

Tea is one of the popular drinks, consumed by a large population of the world. Despite having various health benefits, several studies indicate the toxic effect of tea. In this study, Kulkarni et al. reported a novel extraction procedure through which the toxic effect of tea could be reduced and the presence of kaempferol (osteogenic compound) could be increased by 40-fold. kaempferol enrichment introduced a significant osteogenic effect and reduction of toxic compounds introduced a 20-fold safety pharmacology window. The authors showed that kaempferol-enriched tea not only significantly promoted new bone formation at the fracture site but also prevented bone loss in an osteopenic animal model. This study established the possibility of using kaempferol-enriched tea as a nutraceutical supplement for bone health.

## Use of linagliptin in combination with metformin for diabetic osteoporosis therapy

Diabetes-induced osteoporosis is a growing concern, as this condition is difficult to manage and increases fracture risk greatly. In this study, Nirwan and Vohora demonstrated that the co-treatment strategy of linagliptin along with metformin not only rescued bone microarchitecture but also recovered metabolic parameters like glucose tolerance and lipid profile. Although monotherapy of linagliptin and metformin was previously reported to show osteogenic effects, this study established that a combination of linagliptin and metformin is superior to monotherapy and effective to improve bone microarchitecture, glucose tolerance, lipid profile, and reduce proinflammatory cytokines. This study established the possibility of using linagliptin and metformin combination therapy in diabetic patients with a risk of osteoporosis.

## Using Dieckol, a phyto-constituents of marine brown alga for glucocorticoid-induced osteoporosis therapy

Glucocorticoids are largely used for the treatment of inflammation, pulmonary disorder, immunological conditions, and organ transplantation patients. Prolonged use of glucocorticoids increases the risk of osteoporosis and fracture as a result of high osteoclastic activity and osteocyte apoptosis. In this study, Wang et al. showed that Dieckol, found in the marine brown alga has significant osteogenic potential to counter glucocorticoid-induced osteoporosis. Dieckol treatment not only restored bone microarchitecture but also improved serum biochemical parameters, reduced osteoclastic activity, and increased antioxidant parameters. This study established the possibility of using Dieckol in glucocorticoid-induced osteoporotic condition, although more mechanistic study and safety pharmacology study is required in future.

## Vitamin D supplementation to reduce fall risk, meta-analysis of randomized controlled trials

Falls in older individuals lead to fractures and reduced mobility. Vitamin D is in use for a long time to improve bone health, although various doses of vitamin D and calcium supplementation are used by physicians which can be sometimes confusing. To overcome this limitation, in this systematic review considering 38 randomized controlled trials, Wei et al. found that 700-2000IU of vitamin D supplementation per day was associated with lower fall risk in older adults. The authors also found that a low dose of vitamin D (<700 IU) was not significantly associated with lower fall risk. This meta-analysis will be useful to determine vitamin D supplementation dose in elderly patients.

## Conclusion

Research in the area of Bone biology is a constantly evolving area with a focus to design effective therapeutics for the management of osteoporosis and fracture. I strongly believe that articles published under the Research Topic “*Expert Opinion in Bone Research: 2021*” will help to formulate new strategies for osteoporosis and fracture treatment.

## Author contributions

The author confirms being the sole contributor of this work and has approved it for publication.

## Conflict of interest

The author declares that the research was conducted in the absence of any commercial or financial relationships that could be construed as a potential conflict of interest.

## Publisher’s note

All claims expressed in this article are solely those of the authors and do not necessarily represent those of their affiliated organizations, or those of the publisher, the editors and the reviewers. Any product that may be evaluated in this article, or claim that may be made by its manufacturer, is not guaranteed or endorsed by the publisher.

